# Plasma protein profiling analysis in patients with atrial fibrillation before and after three different ablation techniques

**DOI:** 10.3389/fcvm.2022.1077992

**Published:** 2023-01-10

**Authors:** Menglu Lin, Yangyang Bao, Zunhui Du, Yanting Zhou, Ning Zhang, Changjian Lin, Yinyin Xie, Ruihong Zhang, Qiheng Li, Jinwei Quan, Tingfang Zhu, Yuan Xie, Cathy Xu, Yun Xie, Yue Wei, Qingzhi Luo, Wenqi Pan, Lingjie Wang, Tianyou Ling, Qi Jin, Liqun Wu, Tong Yin, Yucai Xie

**Affiliations:** ^1^Department of Cardiovascular Medicine, Ruijin Hospital, Shanghai Jiao Tong University School of Medicine, Shanghai, China; ^2^State Key Laboratory of Medical Genomics, Shanghai Institute of Hematology, National Research Center for Translational Medicine at Shanghai, Ruijin Hospital, Shanghai Jiao Tong University School of Medicine, Shanghai, China; ^3^College of Osteopathic Medicine, Kansas City University, Kansas City, MO, United States

**Keywords:** proteomics, bioinformatics, Olink, atrial fibrillation, catheter ablation

## Abstract

**Background:**

There are controversies on the pathophysiological alteration in patients with atrial fibrillation (AF) undergoing pulmonary vein isolation using different energy sources.

**Objectives:**

We evaluated the changes in plasma proteins in acute phase post-ablation in patients receiving cryoballoon ablation, radiofrequency balloon ablation, or radiofrequency ablation.

**Methods:**

Blood samples from eight healthy controls and 24 patients with AF were taken on the day of admission, day 1, and day 2 post-ablation and analyzed by the Olink proximity extension assay. Proteins were identified and performed with enrichment analysis. Protein–protein interaction network and module analysis were conducted using Cytoscape software.

**Results:**

Of 181 proteins, 42 proteins in the cryoballoon group, 46 proteins in the radiofrequency balloon group, and 43 proteins in the radiofrequency group significantly changed after ablation. Most of the proteins altered significantly on the first day after ablation. Altered proteins were mainly involved in cytokine–cytokine receptor interaction. Both balloon-based ablations showed a similar shift toward enhancing cell communication and regulation of signaling while inhibiting neutrophil chemotaxis. However, radiofrequency ablation presented a different trend. Seed proteins, including osteopontin, interleukin-6, interleukin-10, C-C motif ligand 8, and matrix metalloproteinase-1, were identified. More significant proteins associated with hemorrhage and coagulation were selected in balloon-based ablations by machine learning.

**Conclusion:**

Plasma protein response after three different ablations in patients with AF mainly occurred on the first day. Radiofrequency balloon ablation shared similar alteration in protein profile as cryoballoon ablation compared with radiofrequency ablation, suggesting that lesion size rather than energy source is the determinant in pathophysiological responses to the ablation.

## Introduction

Atrial fibrillation (AF) is the most common arrhythmia around the world, which can cause a variety of clinical outcomes, such as stroke, heart failure, and cardiovascular mortality. The two main strategies of treatment are anti-arrhythmic drugs and catheter ablation. Although the primary treatment for AF is pharmacology therapy, it has limited effectiveness (1). Previous studies have demonstrated that catheter ablation outperforms drug therapy in terms of recurrence rate and quality of life in long term (2–4). Accordingly, catheter ablation is recommended by the most recent guidelines (5, 6).

Currently, radiofrequency (RF) ablation and cryoballoon (CB) ablation are the most common techniques to treat AF, with no significant difference in terms of safety or efficacy (7, 8). Experimental data suggested that the use of cryoablation is different from radiofrequency ablation due to its preservation of the extracellular matrix and reduction of endothelial and thrombus formation (9–11). In clinical studies, however, there was a comparable increase in markers of cell damage, platelet activation, and inflammatory response between CB and RF ablation (12, 13). Radiofrequency balloon (RB) ablation, also called hot balloon ablation, is an emerging approach for AF ablation with favorable safety and effectiveness (14, 15). However, there are few data about the biomarkers’ changes compared to the CB or RF ablation.

In order to resolve the controversy, we utilized a new proteomic assay platform—Olink proximity extension assay, which has been widely applied in the biomarker discovery of the cardiovascular disease field (16, 17), to assess the change in 181 different proteins during the acute post-procedure phase from the cryoballoon ablation group and irrigated-tip RF catheter ablation group. We also introduced the RB ablation group into comparison to avoid alteration resulting from lesion size rather than energy sources (18).

## Materials and methods

### Patient samples

After the Institutional Review Committee and Ethics Committee approved the study, 24 patients diagnosed with paroxysmal or persistent AF at our institution who gave informed written consent were included, in which pulmonary vein isolation was performed with the CB catheter (Arctic Front Advance Cardiac Cryoablation Catheter, Medtronic), RB catheter (HELIOSTAR™ Balloon Ablation Catheter, Biosense Webster Inc.), or RF catheter (NaviStar™RMT, ThermoCool™; Biosense Webster, CA, USA). In our study, all patients met the following inclusion criteria: (1) Non-valvular AF or flutter. The exclusion criteria were as follows: (1) <18 or >80 years old; (2) history of left atrial surgery; (3) transesophageal echocardiography-witnessed left atrial thrombus; (4) uncontrolled hyperthyroidism; (5) pregnancy; (6) history of obstructive sleep apnea syndrome; and (7) history of myocardial infarction, percutaneous coronary intervention, heart surgery, transient ischemic attack, or stroke within 3 months before the procedure. Patients were treated with oral anticoagulation at least 1 month before the ablation procedure. During the procedure, 100-IU/kg heparin was administered to maintain an activated clotting time of ≥300 s. Eight healthy controls were also enrolled as a comparison. A blood sample of 2 ml from the peripheral vein was taken and added to the ethylene diamine tetraacetic acid (EDTA) anticoagulant tube on three-time points, the admission day, day 1 (D1), and day 2 (D2) after ablation. The samples were immediately centrifuged and frozen at −80°C until analysis. All the patients were monitored by the telemetry electrocardiogram during hospitalization, and electrocardiogram data were collected for analysis.

### Olink proteomic profiling

The proteomic analyses were performed at the National Research Center for Translational Medicine, Ruijin Hospital, Shanghai Jiao Tong University School of Medicine. The Olink multiplex cardiovascular disease III and inflammation panels comprise 184 proteins. Three protein markers were available on both panels, resulting in 181 unique markers. Samples were run in duplicates. The limit of detection (LOD) was defined as three standard deviations above the background, and values below this limit were reported as <LOD. The results from each assay were rendered as normalized log_2_ protein expression (NPX) data, which are relative values, and one-unit higher NPX represents a doubling of the measured protein concentration. NPX values for proteins with >75% of measurements below LOD were removed from the analysis.

### Statistic analysis

Statistical analysis was performed using GraphPad Prism nine software and SPSS 25. Normally distributed continuous variables were shown as mean ± SD. Categorical variables were shown as counts and percentages. Categorical variables were compared with the chi-square test. Wilcoxon matched-pairs signed-rank test was used to assess differences for each analyte before and after ablation. The Kruskal–Wallis *H*-test was used to assess differences in the clinical characteristics and the levels of analytes according to the ablation type. A *P*-value of <0.05 was considered statistically significant.

### Proteins bioinformatic analysis

Heatmap was plotted by http://www.bioinformatics.com.cn, a free online platform for data analysis and visualization. The protein interaction, the gene ontology (GO) functional, and Kyoto Encyclopedia of Genes and Genomes (KEGG) pathway enrichment analysis in the network were performed by Search Tool for the Retrieval of Interacting Genes (STRING, version 11.5) database with the threshold of false discovery rate (FDR) <0.05. The required confidence (combined score) >0.4 was used as the cutoff criterion. In addition, the Molecular Complex Detection (MCODE) plugin in Cytoscape (version 3.7.1) was used to screen the significant modules and seed protein in the protein–protein interaction (PPI) network (degree cutoff = 2, node cutoff = 0.2, k-core = 2, and max. depth = 100). Moreover, enrichment analysis for the proteins involved in the most significant module was conducted using the STRING online tool. LASSO logistic regression model was fitted with the glmnet R package. Feature contributions to the models were visualized using the regression coefficients. The random forest model was fitted with random Forest and caret R packages according to Jack Gisby et al. with slight modifications (19). The 4-fold cross-validation that was repeated 100 times was used to estimate the model accuracy. Mtry value was calculated as the square root of the number of features. The random forest feature was extracted using the R randomForestExplainer package. Benjamini–Hochberg adjustment was applied to the *p*-value calculated by the model, and an adjusted value of 0.05 was indicated as the significance threshold. Multiway importance plot was replotted *via* R package ggplot2.

## Results

### Baseline characteristics and clinical outcomes after ablation

A total of 32 persons were included in this study, of which eight were controls and eight received CB, RB, or RF ablation, respectively. The baseline characteristics of 32 participants are presented in Table 1. There were no significant differences in ages, gender, laboratory values, past medical history, and the history of medication apart from the use of anticoagulants (*p* < 0.01).

**TABLE 1:** Clinical characteristics at baseline.

	Controls (*n* = 8)	Cryoballoon (*n* = 8)	Radiofrequency (*n* = 8)	Radiofrequency balloon (*n* = 8)	*P*-value
Age (years)	65.13 ± 3.56	65.63 ± 3.25	63.50 ± 1.69	58.63 ± 4.13	0.610
Male (*n*%)	5 (62.5%)	4 (50%)	3 (37.5%)	4 (50%)	0.801
BMI (kg/m2)	22.13 ± 0.92	22.96 ± 0.87	24.07 ± 0.77	23.73 ± 0.83	0.373
Smoking history (*n*%)	3 (37.5%)	2 (25%)	0 (0%)	2 (25%)	0.324
Alcohol history (*n*%)	1 (12.5%)	1 (12.5%)	0 (0%)	2 (25%)	0.515
Past medical history					
Hypertension (*n*%)	4 (50%)	2 (25%)	4 (50%)	4 (50%)	0.677
Diabetes (*n*%)	0 (0%)	0 (0%)	2 (25%)	1 (12.5%)	0.257
Coronary heart disease (*n*%)	1 (12.5%)	0 (0%)	1 (12.5%)	1 (12.5%)	0.776
Cerebral ischemia (*n*%)	2 (25%)	1 (12.5%)	0 (0%)	1 (12.5%)	0.515
Peripheral vascular disease (*n*%)	0 (0%)	0 (0%)	0 (0%)	0 (0%)	/
CHA_2_DS_2_-VASc score					
Score ≤1 (*n*%)	/	5 (62.5%)	2 (25%)	4 (50%)	/
Score ≥2 (*n*%)	/	3 (37.5%)	6 (75%)	4 (50%)	/
Atrial fibrillation type					
Paroxysmal (*n*%)	/	6 (75%)	5 (62.5%)	8 (100%)	/
Persistent (*n*%)	/	2 (25%)	3 (37.5%)	0 (0%)	/
Atrial fibrillation duration (months)	/	26.63 ± 17.43	15 ± 13.29	13.25 ± 8.54	/
Echocardiography					
Left atrium diameter (mm)	37.63 ± 0.56	40.00 ± 1.18	40.50 ± 1.31	38.13 ± 1.69	0.095
Ejection fraction (%)	64.50 ± 1.54	68.88 ± 1.29	66.00 ± 1.52	66.00 ± 1.52	0.223
Transesophageal echocardiography					
Normal (*n*%)	8 (100%)	7 (87.5%)	6 (75%)	7 (87.5%)	0.515
Dense echo (*n*%)	0 (0%)	1 (12.5%)	2 (25%)	1 (12.5%)	0.515
Thrombus (*n*%)	0 (0%)	0 (0%)	0 (0%)	0 (0%)	/
Thrombus in other places (*n*%)	0 (0%)	0 (0%)	0 (0%)	0 (0%)	/
Medicine history					
Beta blockers (*n*%)	2 (25%)	3 (37.5%)	1 (12.5%)	3 (37.5%)	0.637
alpha blockers (*n*%)	1 (12.5%)	0 (0%)	0 (0%)	0 (0%)	0.377
ACEI/ARB (*n*%)	2 (25%)	1 (12.5%)	2 (25%)	2 (25%)	0.908
Diuretics (*n*%)	1 (12.5%)	0 (0%)	1 (12.5%)	1 (12.5%)	0.776
Ca2 + antagonist (*n*%)	2 (25%)	2 (25%)	1 (12.5%)	1 (12.5%)	0.845
Digitalis (*n*%)	0 (0%)	0 (0%)	0 (0%)	0 (0%)	/
Antiarrhythmic drugs (*n*%)	0 (0%)	2 (25%)	3 (37.5%)	4 (50%)	0.144
Antiplatelet drugs (*n*%)	2 (25%)	0 (0%)	0 (0%)	0 (0%)	0.094
Anticoagulant (*n*%)	0 (0%)	8 (100%)	8 (100%)	8 (100%)	<0.01
Lab results					
WBC (*10^9/L)	5.05 ± 0.55	5.13 ± 0.33	5.61 ± 0.49	5.89 ± 0.98	0.646
RBC (*10^12/L)	4.30 ± 0.11	4.55 ± 0.14	4.43 ± 0.15	4.34 ± 0.11	0.574
Platelet (*10^9/L)	185.63 ± 14.16	198.13 ± 18.96	207.75 ± 19.45	206.63 ± 20.82	0.924
Hb (g/L)	134.25 ± 3.82	139.38 ± 4.44	136.13 ± 5.63	133.88 ± 3.20	0.862
ALT (IU/L)	16.13 ± 1.88	31.25 ± 8.38	24.63 ± 3.24	24.88 ± 5.83	0.151
AST (IU/L)	21.38 ± 0.91	30.50 ± 8.64	21.63 ± 1.52	25.13 ± 6.25	0.903
Cr (μml/L)	78.75 ± 4.83	74.75 ± 6.70	68.25 ± 3.07	70.50 ± 5.62	0.504
TC (mmol/L)	4.41 ± 0.28	4.14 ± 0.35	4.58 ± 0.28	4.13 ± 0.20	0.654
LDL (mmol/L)	2.55 ± 0.36	2.36 ± 0.32	2.76 ± 0.16	2.53 ± 0.19	0.804
HbA1c (%)	5.48 ± 0.09	5.83 ± 0.17	6.43 ± 0.59	5.55 ± 0.17	0.401
pro-BNP (pg/ml)	75.56 ± 29.57	359.54 ± 196.27	256.65 ± 84.54	312.08 ± 226.52	0.186
TnI (ng/ml)	0.39 ± 0.37	0.01 ± 0.00	0.66 ± 0.48	0.01 ± 0.00	0.217
D-dimer (mg/L)	0.23 ± 0.04	0.24 ± 0.03	0.50 ± 0.32	0.30 ± 0.02	0.073

Continuous variables were shown as mean ± SD. Categorical variables were shown as counts and percentages. N, number; BMI, body mass index; CHA_2_DS_2_-VASc, risk score for thromboembolism; ACEI, angiotensin-converting enzyme inhibitors; ARB, angiotensin receptor blockers; WBC, white blood cells; RBC, red blood cells; Hb, hemoglobin; ALT, alanine transaminase; AST, aspartate transaminase; Cr, creatine; TC, total cholesterol; LDL, low-density lipoprotein; HbA1c, glycated hemoglobin; BNP, brain natriuretic peptides; TnI, troponin I.

**FIGURE 1:**
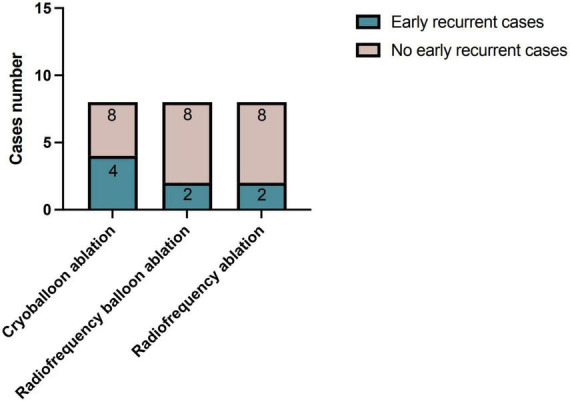
Early recurrence of atrial arrhythmia post-different ablations during hospitalization. The early recurrent cases of atrial arrhythmia post-ablations were not significantly different between three ablation groups (*p* = 0.472).

All the patients were monitored by the telemetry electrocardiogram during the hospitalization. The recurrent atrial arrhythmia after ablation has no significant differences in the three groups, although four patients presented AF or premature atrial contraction in the CB group, compared with two patients in the RB and RF groups, respectively (*p* = 0.472, Figure 1).

### The change in plasma proteins before and after three different ablations

A total of 181 unique proteins in two panels were tested, and 19 (10.5%) analytes were removed with over 75% of samples below LOD. Table 2 shows the biomarkers that showed significant differences before and after ablation using three different methods. Forty-two proteins (19 increased and 24 decreased), 46 proteins (21 increased and 25 decreased), and 43 proteins (13 increased and 30 decreased) showed significant changes in the CB, RB, and RF groups, respectively (Supplementary Figure 4).

**TABLE 2:** Significantly altered proteins before and after ablation were sorted by three different techniques.

Cryoballoon ablation	
Increased proteins	LDL receptor, MMP-9, GDF-15, PRTN3, OPN, CHI3L1, ST2, IGFBP-2, MB (D1), IL-8, TFG-alpha, IL-10, CCL23, CSF-1, MCP-3, IL-17C, CCL20, IL-6, and OSM
Decreased proteins	IGFBP-1, Ep-CAM, CXCL11, CXCL9, MCP-4, CCL11, CCL19, TRANCE, IL-12B, Flt3L, IFN-gamma, CCL8, CD244, CD6, FGF-21, TNFRSF9, uPA, MCP-1, TRAIL, CST5, CXCL10, CCL25, and TNFB, MB (D2)
Radiofrequency balloon ablation	
Increased proteins	MMP-9, MB, CHI3L1, ST2, IGFBP-2, TNFRSF14, LDL receptor, EPHB4, GRN, LTBR, TR, IL-1RT1, CTSD, CTSZ, TNF-R1, OPN, IL-6, OSM, CASP-8, TNFSF14, and CSF-1
Decreased proteins	TFF3, CSTB, MCP-1, uPA, COL1A1, vWF, TRAIL, CXCL9, CD6, CCL11, MMP-1, CCL19, TRANCE, Flt3L, CXCL6, CXCL10, CCL28, IFN-gamma, MCP-2, TNFB, IL-8, TWEAK, CXCL11, CST5, and MCP-4
Radiofrequency ablation	
Increased proteins	GDF-15, RETN, TR-AP, PRTN3, OPN, ST2, IGFBP-2, CHI3L1, IL-10, MCP-3, CSF-1, IL-6, and CCL23
Decreased proteins	CCL15, uPA, CPB1, MMP-3, CSTB, CD163, BLM hydrolase, IGFBP-1, Ep-CAM, Gal-4, KLK6, PLC, CDH5, FABP4, COL1A1, GDNF, TRAIL, CST5, SLAMF1, CCL11, MMP-1, CCL19, TRANCE, MMP-10, Flt3L, IFN-gamma, CCL25, TWEAK, TNFB, and FGF-21

**FIGURE 2:**
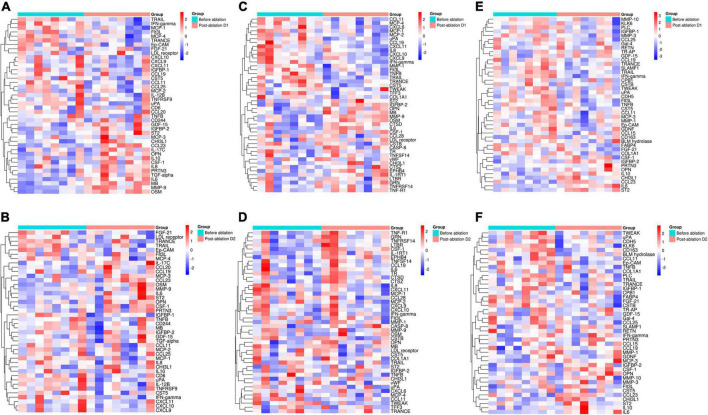
Heatmap of altered proteins before and after ablation. Most of the altered proteins changed on the first day after ablation. The heatmap of the altered proteins before and after ablation D1 and D2 in the cryoballoon group **(A,B)**, the radiofrequency balloon group **(C,D)**, and the radiofrequency group **(E,F)** (red: high expression and blue: low expression).

The heatmaps of the altered proteins in three groups before and after ablation are shown in Figure 2, which indicates that the change in proteins in the acute phase was mainly on D1 post-ablation, especially in CB and RB groups.

### Enrichment analysis and module analysis of PPI network in three ablation groups

**FIGURE 3:**
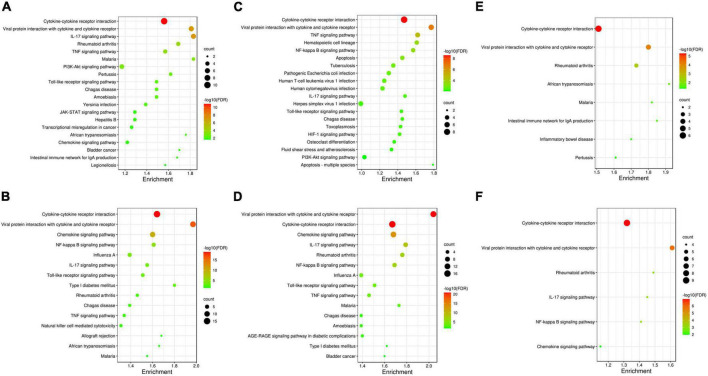
Kyoto encyclopedia of genes and genomes pathways analysis of altered proteins in three ablation groups. Altered proteins in three groups are all mainly enriched in cytokine–cytokine receptor interaction and viral protein interaction with cytokine and cytokine receptor. **(A,B)** The top 20 KEGG pathways enriched for the increased proteins and decreased proteins in the cryoballoon ablation group; **(C,D)** the top 20 KEGG pathways enriched for the increased proteins and decreased proteins in the radiofrequency balloon ablation group; **(E,F)** the all KEGG pathways enriched for the increased proteins and decreased proteins in the radiofrequency ablation group. The sizes of the bubbles are illustrated from big to small in descending order of the number of potential targets involved in the pathways.

Based on the altered proteins and the STRING database, we constructed the PPI network for the increased and decreased proteins separately in three groups (Supplementary Figures 1–3). The top KEGG pathways of altered proteins in different groups are shown in Figure 3. There was no distinguishment because the altered proteins in three groups were mainly enriched in cytokine–cytokine receptor interaction and viral protein interaction with cytokine and cytokine receptor.

**FIGURE 4:**
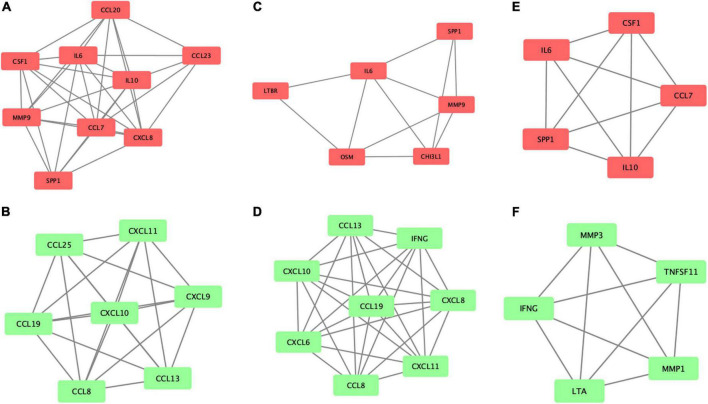
Potential functional modules with the best score in the PPI network. The most significant module for the increased proteins **(A)** and decreased proteins **(B)** in the cryoballoon group; **(C,D)** in the radiofrequency balloon group and **(E,F)** in the radiofrequency group, respectively. The significant modules were analyzed by using MCODE plugin, and then module networks were visualized using Cytoscape software.

In order to more accurately elucidate the protein change pattern after different ablations, we used the MCODE plugin in the Cytoscape software to conduct module analysis on the PPI network and obtained a potential protein functional module with the best score, as shown in Figure 4. Furthermore, the GO enrichment analysis for six significant modules (Supplementary Tables 1–5) showed a similar biological process in CB and RB groups. Proteins involved in the most significant module for the decreased proteins were both enriched in the neutrophil chemotaxis (Tables 3, 4). Proteins in the most significant module for the increased proteins in the CB group mainly enriched in the cytokine-mediated signaling pathway (Table 3), which is part of cell communication and regulation of signaling, the two major positively regulated biological processes in the RB group (Table 4). However, as seen in Table 5, the cytokine-mediated signaling pathway was downregulated in the RF group.

**TABLE 3:** GO_BP and KEGG pathways enriched in the most significant module in the CB group (only top five listed).

Category	Term	Count	FDR	Matching proteins in the most significant module
(a)				
GO_BP	GO: 0019221 Cytokine-mediated signaling pathway GO: 0030595 Leukocyte chemotaxis GO: 0006954 Inflammatory response GO: 0097529 Myeloid leukocyte migration GO: 0002687 Positive regulation of leukocyte migration	8 6 7 5 5	1.79E-07 1.79E-07 1.02E-06 2.50E-06 4.19E-06	CXCL8, CSF-1, CCL20, MMP-9, CCL7, IL-6, IL-10, CCL23 CXCL8, CCL20, CCL7, IL-6, IL-10, CCL23 CXCL8, CSF-1, CCL20, CCL7, SPP1, IL-6, CCL23 CXCL8, CCL20, CCL7, IL-6, CCL23 CXCL8, CSF-1, CCL20, CCL7, IL-6
PATHWAYS	has04061 Viral protein interaction with cytokine and cytokine receptor has04657 IL-17 signaling pathway has04060 Cytokine-cytokine receptor interaction has05323 Rheumatoid arthritis has04668 TNF signaling pathway	7 7 7 7 6	2.05E-13 2.05E-13 2.05E-13 3.89E-13 2.87E-12	CCL13, CXCL10, CXCL11, CCL19, CXCL9, CCL25, CCL8 CCL13, CXCL10, CXCL11, CCL19, CXCL9, CCL25, CCL8 CCL13, CXCL10, CXCL11, CCL19, CXCL9, CCL25, CCL8 CCL13, CXCL10, CXCL11, CCL19, CXCL9, CCL25, CCL8 CCL13, CXCL10, CXCL11, CCL19, CCL25, CCL8
(b)				
GO_BP	GO: 0030593 Neutrophil chemotaxis GO: 0031640 Killing of cells of other organism GO: 0070098 Chemokine-mediated signaling pathway GO: 0061844 Antimicrobial humoral immune response mediated by antimicrobial peptide GO: 0048247 Lymphocyte chemotaxis	7 7 7 7 6	2.05E-13 2.05E-13 2.05E-13 3.89E-13 2.87E-12	CCL13, CXCL10, CXCL11, CCL19, CXCL9, CCL25, CCL8 CCL13, CXCL10, CXCL11, CCL19, CXCL9, CCL25, CCL8 CCL13, CXCL10, CXCL11, CCL19, CXCL9, CCL25, CCL8 CCL13, CXCL10, CXCL11, CCL19, CXCL9, CCL25, CCL8 CCL13, CXCL10, CXCL11, CCL19, CCL25, CCL8
PATHWAYS	has04061 Viral protein interaction with cytokine and cytokine receptor has04062 Chemokine signaling pathway has04060 Cytokine-cytokine receptor interaction has04620 Toll-like receptor signaling pathway has04064 NF-kappa B signaling pathway	7 7 7 3 2	3.05E-14 1.37E-12 1.59E-11 0.00042 0.038	CCL13, CXCL10, CXCL11, CCL19, CXCL9, CCL25, CCL8 CCL13, CXCL10, CXCL11, CCL19, CXCL9, CCL25, CCL8 CCL13, CXCL10, CXCL11, CCL19, CXCL9, CCL25, CCL8 CXCL10, CXCL11, CXCL9 CCL13, CCL19

(a) The most significant module of increased proteins in cryoballoon ablation; (b) the most significant module of decreased proteins in cryoballoon ablation. CB, cryoballoon; GO, gene ontology; BP, biological process; KEGG, Kyoto encyclopedia of genes and genomes; FDR, false-discovery rate.

**TABLE 4:** GO_BP and KEGG pathways enriched in the most significant module in the RB group (only top five listed).

Category	Term	Count	FDR	Matching proteins in the most significant module
(a)				
GO_BP	GO: 0010647 Positive regulation of cell communication GO: 0023056 Positive regulation of signaling GO: 0001934 Positive regulation of protein phosphorylation GO: 0071310 Cellular response to organic substance GO: 0071345 Cellular response to cytokine stimulus	6 6 5 6 5	0.0085 0.0085 0.0093 0.0093 0.0093	OSM, LTBR, CHI3L1, MMP-9, SPP1, IL-6 OSM, LTBR, CHI3L1, MMP-9, SPP1, IL-6 OSM, LTBR, CHI3L1, MMP-9, IL-6 OSM, LTBR, CHI3L1, MMP-9, SPP1, IL-6 OSM, LTBR, CHI3L1, MMP-9, IL-6
PATHWAYS	has04060 Cytokine-cytokine receptor interaction has04151 PI3K-Akt signaling pathway has04672 Intestinal immune network for IgA production has04061 Viral protein interaction with cytokine and cytokine receptor has04066 HIF-1 signaling pathway	3 3 2 2 2	0.0199 0.0199 0.0199 0.0284 0.0284	OSM, LTBR, IL-6 OSM, SPP1, IL-6 LTBR, IL-6 LTBR, IL-6 LTBR, IL-6
(b)				
GO_BP	GO: 0030593 Neutrophil chemotaxis GO: 0070098 Chemokine-mediated signaling pathway GO: 0006959 Humoral immune response GO: 0061844 Antimicrobial humoral immune response mediated by antimicrobial peptide GO: 0006954 Inflammatory response	7 7 8 7 8	1.63E-12 1.63E-12 2.78E-12 3.10E-12 2.26E-10	CCL13, CXCL6, CXCL10, CXCL8, CXCL11, CCL19, CCL8 CCL13, CXCL6, CXCL10, CXCL8, CXCL11, CCL19, CCL8 CCL13, CXCL6, IFNG, CXCL10, CXCL8, CXCL11, CCL19, CCL8 CCL13, CXCL6, CXCL10, CXCL8, CXCL11, CCL19, CCL8 CCL13, CXCL6, IFNG, CXCL10, CXCL8, CXCL11, CCL19, CCL8
PATHWAYS	has04061 Viral protein interaction with cytokine and cytokine receptor has04060 Cytokine-cytokine receptor interaction has04062 Chemokine signaling pathway has04657 IL-17 signaling pathway has05323 Rheumatoid arthritis	7 8 7 4 3	2.43E-13 3.54E-13 7.22E-12 3.15E-06 0.00033	CCL13, CXCL6, CXCL10, CXCL8, CXCL11, CCL19, CCL8 CCL13, CXCL6, IFNG, CXCL10, CXCL8, CXCL11, CCL19, CCL8 CCL13, CXCL6, CXCL10, CXCL8, CXCL11, CCL19, CCL8 CXCL6, IFNG, CXCL10, CXCL8 CXCL6, IFNG, CXCL8

(a) The most significant module of increased proteins in radiofrequency balloon ablation; (b) the most significant module of decreased proteins in radiofrequency balloon ablation. RB, radiofrequency balloon; GO, gene ontology; BP, biological process; KEGG, kyoto encyclopedia of genes and genomes; FDR, false-discovery rate.

**TABLE 5:** GO_BP and KEGG pathways enriched in the most significant module in the RF group (only top five listed).

Category	Term	Count	FDR	Matching proteins in the most significant module
(a)				
GO_BP	GO: 0032101 Regulation of response to external stimulus GO: 0022603 Regulation of anatomical structure morphogenesis GO: 0002690 Positive regulation of leukocyte chemotaxis GO: 0006952 Defense response GO: 0006954 Inflammatory response	5 5 3 5 4	0.0048 0.0048 0.0055 0.0055 0.0062	CSF-1, CCL7, SPP1, IL-6, IL-10 CSF-1, CCL7, SPP1, IL-6, IL-10 CSF-1, CCL7, IL-6 CSF-1, CCL7, SPP1, IL-6, IL-10 CSF-1, CCL7, SPP1, IL-6
PATHWAYS	has04061 Viral protein interaction with cytokine and cytokine receptor has04060 Cytokine-cytokine receptor interaction has05143 African trypanosomiasis has04151 PI3K-Akt signaling pathway has04672 Intestinal immune network for IgA production	4 4 2 3 2	1.07E-06 3.71E-05 0.0041 0.0043 0.0043	CSF-1, CCL7, IL-6, IL-10 CSF-1, CCL7, IL-6, IL-10 IL-6, IL-10 CSF-1, SPP1, IL-6 IL-6, IL-10
(b)				
GO_BP	GO: 0019221 Cytokine-mediated signaling pathway GO: 0050729 Positive regulation of inflammatory response	5 3	0.00066 0.0132	IFNG, MMP-3, MMP-1, TNFSF11, LTA IFNG, TNFSF11, LTA
PATHWAYS	has05323 Rheumatoid arthritis has04657 IL-17 signaling pathway has04060 Cytokine-cytokine receptor interaction has04940 Type I diabetes mellitus has04064 NF-kappa B signaling pathway	4 3 3 2 2	6.69E-07 0.00018 0.0033 0.0036 0.0182	IFNG, MMP-3, MMP-1, TNFSF11 IFNG, MMP-3, MMP-1 IFNG, TNFSF11, LTA IFNG, LTA TNFSF11, LTA

(a) The most significant module of increased proteins in radiofrequency ablation; (b) the most significant module of decreased proteins in radiofrequency ablation. RF, radiofrequency; GO, gene ontology; BP, biological process; KEGG, kyoto encyclopedia of genes and genomes; FDR, false-discovery rate.

### Identification of the seed protein under three different techniques

The MCODE application also provided the highest dense protein complex as the seed protein from the potential functional module, which helps to identify the potential markers for further study in three different techniques. The seed protein of the increased proteins was osteopontin (OPN), also known as secreted phosphoprotein 1, in the CB group and interleukin-6 (IL-6) in the RB group. For patients who received RF ablation, the seed increased protein was IL-10.

Among the proteins decreased after ablation, the seed protein was C-C motif ligand 8 (CCL8), also known as monocyte chemoattractant protein-2, in both CB and RB groups. Differently, the seed protein was matrix metalloproteinase-1 (MMP-1) in the RF group.

**FIGURE 5:**
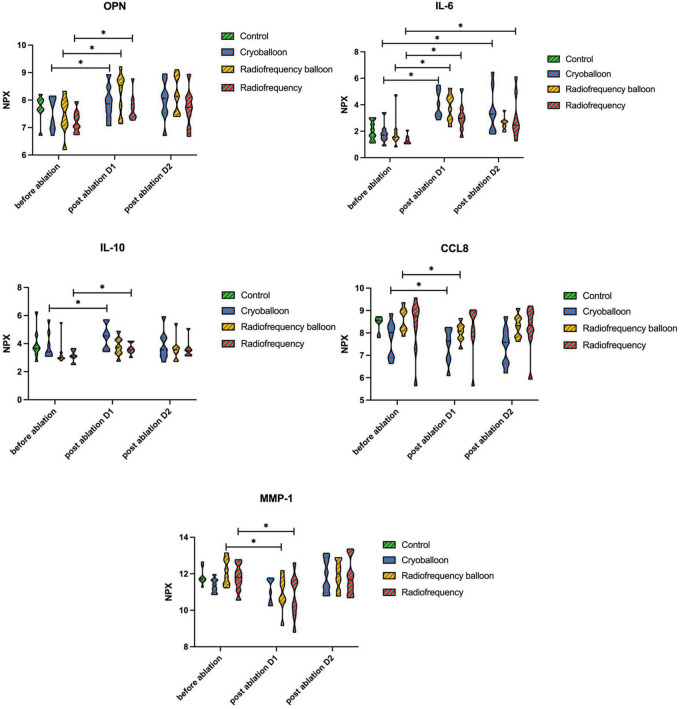
Levels of seed proteins before and after ablation. No differences were seen between the groups at any time point. Differences before and after ablation were assessed with the Wilcoxon signed-rank test. *P*-values are given for comparison within the group between different time points. OPN, osteopontin, seed-increased protein in the CB group; IL-6, interleukin-6, seed-increased protein in the RB group; IL-10, interleukin-10, seed-increased protein in the RF group; CCL8, C-C motif ligand 8, seed decreased protein in CB and RB groups; MMP-1, matrix metalloproteinase-1, seed decreased protein in the RF group. NPX, normalized protein expression. *P < 0.05.

The change in OPN, IL-6, IL-10, CCL8, and MMP-1 levels at three-time points in three ablation groups and the control group are shown in Figure 5, with a comparable measurement between the groups. No significant difference was observed in these proteins at baseline between the control group and three ablation groups as well, which can exclude the disease-related effect on the change in proteins.

### Biomarkers associated with hemorrhage and coagulation activity

**FIGURE 6:**
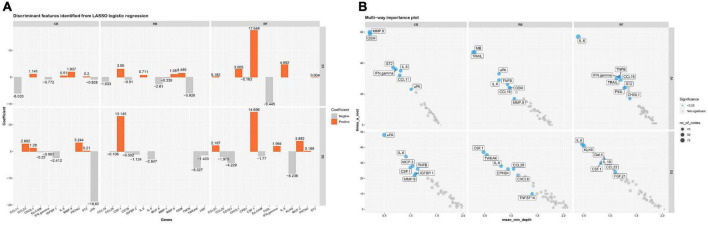
LASSO logistic regression and random forest of altered proteins in three ablation groups. **(A)** Significant proteins selected by LASSO logistic regression before and after ablation D1 and D2 in three ablation groups; **(B)** significant proteins selected by random forest before and after ablation D1 and D2 in three ablation groups.

**FIGURE 7:**
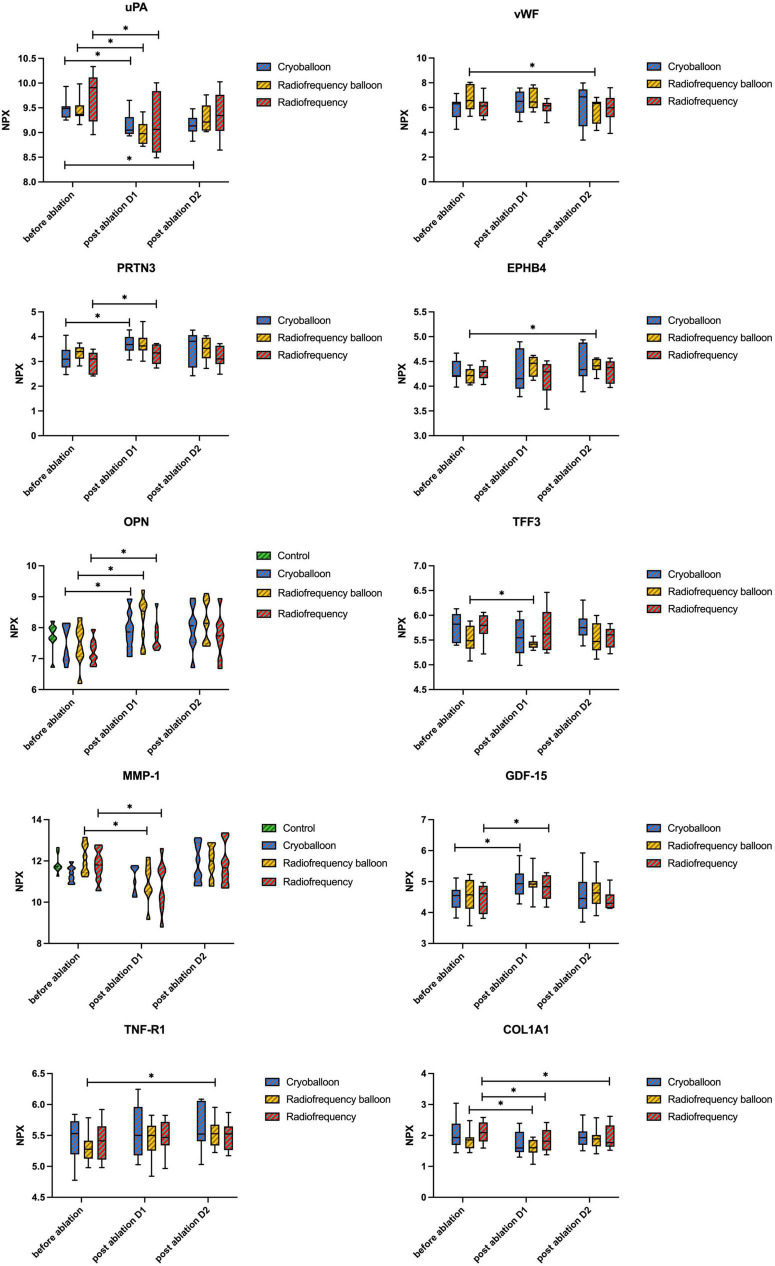
Levels of proteins associated with hemorrhage and coagulation activity. No differences were seen between the groups at any time point. Differences before and after ablation were assessed with the Wilcoxon signed-rank test. *P*-values are given for comparison within the group between different time points. uPA, urokinase-type plasminogen activator; TFF3, trefoil factor 3; GDF-15, growth/differentiation factor 15; PRTN3, myeloblastin; COL1A1, collagen alpha-1(I) chain; TNF-R1, tumor necrosis factor receptor 1; EPHB4, ephrin type-B receptor 4; vWF, von Willebrand factor; NPX, normalized protein expression; *P < 0.05. Note that the levels of OPN and MMP-1 are shown in Figure 5.

As shown in Table 6, we identified some altered proteins associated with hemorrhage and coagulation. Proteins changes that increased thrombus formation risk after ablation were noted for OPN and urokinase-type plasminogen activator (uPA) in all groups, along with trefoil factor 3 (TFF3) in the RB group and MMP-1 in the RF group. Proteins changes that caused higher bleeding risk after ablation, include growth/differentiation factor 15, myeloblastin (PRTN3), collagen alpha-1 (I) chain, tumor necrosis factor receptor 1 (TNF-R1), ephrin type-B receptor 4 (EPHB4), and von Willebrand factor (vWF). We also conducted LASSO logistic regression and random forest to select significant proteins (Figure 6). Interestingly, two proteins (uPA and PRTN3) in the CB group and three proteins (vWF, uPA, and EPHB4) in the RB group were also the significant proteins selected by LASSO logistic regression and random forest, among which only PRTN3 was related to hemorrhage and coagulation in the RF group. No significant differences in the levels of these proteins were found between three groups (Figure 7).

**TABLE 6:** Protein changes are associated with hemorrhage and coagulation activity.

Group	Increase thrombus risk	Increase bleeding risk
Cryoballoon ablation	OPN↑, uPA↓	GDF-15↑, PRTN3↓
Radiofrequency balloon ablation	OPN↑, uPA↓, TFF3↓	TNF-R1↑, EPHB4↑, COL1A1↓, vWF↓
Radiofrequency ablation	OPN↑, uPA↓, MMP-1↓	GDF-15↑, PRTN3↑, COL1A1↓

↑, proteins increased after ablation; ↓, proteins decreased after ablation.

## Discussion

### Proteins mainly altered on the first day after ablation

In this study, we analyzed a total of 181 different plasma proteins from eight control patients and 24 patients with AF before and after interventional ablation using three different techniques. Most of the proteins changed significantly on the first day after ablation. Although clinical studies demonstrated that same-day discharge is safe with a comparable rate of complications in the long term in patients with an overnight stay after ablation (20, 21), the pathophysiological alterations recover until the overnight stay in patients with AF undergoing pulmonary vein isolation. Given that plasma proteins changed mainly limited on the first day after three different ablations, it would be safer for patients to be discharged overnight.

### Similar enrichment analysis in both balloon-based ablations

Pathway analysis indicated that the proteins that changed after ablation in the acute phase were mainly in cytokine–cytokine receptor interaction in three groups. Interestingly, the module analysis and enrichment analysis indicated that CB and RB groups showed similar trends in cell communication, the regulation of signaling, and neutrophil chemotaxis. Specifically, the cytokine-mediated signaling pathway in the CB group was upregulated significantly. As part of cell communication and regulation of signaling, this signaling pathway was also positively regulated in the RB group (Supplementary Table 3). However, the cytokine-mediated signaling pathway was downregulated in the RF group. Meanwhile, neutrophil chemotaxis was inhibited in both balloon-based ablations (Tables 3, 4) but not in the RF group. To our knowledge, this phenomenon was not noted in previous studies.

### Similar response to inflammation in both balloon-based ablations

We also found that both balloon-based ablations shared a similar response to inflammation, while radiofrequency ablation showed different tendencies to inflammatory response as indicated by different seed proteins after ablation. In the CB group, OPN was the most important increased protein after ablation. OPN is a matricellular protein that contributes to acute inflammation by increasing the production of IL-6, IL-12, IL-17, and interferon-gamma (IFN-γ) and inhibiting the expression of IL-10 (22). Since RB ablation is a new technique, there are few comparative studies about markers of inflammation between RB and CB ablation or RF ablation. However, the high risk of inflammation in the RB group was indicated by the seed protein IL-6 in our study. Such a finding was consistent with a previous image study, which describes the regional inflammation pattern occurring after CB ablation and RB ablation in the human left atrium using positron emission tomography (23). The upregulated expression of IL-6 by OPN has been demonstrated in inflammatory disease (24). Thus, IL-6 may trigger IL-6 trans-signaling, contributing to the upregulation of OPN in THP-1 macrophages (25). Of note, higher IL-6 concentrations can also play a role in regulating leukocyte infiltration by suppressing neutrophil recruitment (26). Neutrophil activation, as reflected by the seed protein CCL8 (27, 28), displayed a similar decreased trend in the CB and RB group in this study. While in the RF group, IL-10 generally recognized as an anti-inflammatory cytokine, was the seed protein of increased proteins in our study. The new report demonstrated that mature neutrophils could potentially dampen local inflammation by IL-10 production (29).

There is no obvious explanation for why these proteins altered significantly, but it could be related to larger lesion size and injury. Previous studies have shown that endothelial injury is related to inflammatory biomarkers, including OPN and IL-6 (30, 31). High levels of IL-6 are also associated with vascularization in sites of inflammation and with wound healing (32, 33). The higher inflammatory tendency in balloon-based ablation might show that the endothelial surface is kept less intact after balloon lesion than what is seen after irrigated RF ablation.

Although this study noted comparable levels of inflammatory markers in the acute phase between the CB and RF ablation, which is supported by other publications (34, 35), the different inflammatory effects of seed proteins indicates greater local inflammatory response in the balloon-based ablation. Along with similar enrichment analysis, these results suggest that pathophysiological response in the acute phase after ablation is more likely to be affected by the lesion size rather than the energy source.

### Proteins associated with hemorrhage and coagulation activity in three ablations

Thromboembolism events are uncommon but one of the most feared complications of AF ablation. By using a new proteomic assay, we identified some proteins which are different from the common-used biomarkers in previous studies, such as D-dimer and P-selection.

As shown in Table 6, radiofrequency energy affected greater numbers of proteins, indicating a stronger impact on hemorrhage and coagulation activity. This finding is consistent with previous studies showing a higher rate of thrombus formation after RF than after CB ablation (11, 36). However, current data are lacking to define the prevalence of thromboembolism in patients received RB ablation. Compared with the CB group, both radiofrequency energy groups affected one more protein that could increase the risk of thrombus. As the most important decreased protein in the RF group in our study, MMP-1 plays an important role in the occurrence and development of a deep venous thrombus (37, 38), and also in regulating prothrombotic state in patients with AF (39). TFF3 is a protective marker of underlying myocyte damage/ischemia and is moderately correlated with ischemic stroke in patients with AF (40, 41). We also noticed the bleeding tendency with the lower level of vWF after RB ablation, which is commonly increased after CB and RF ablation (13, 42). In an explorative study, TNF-R1 and EPHB4 were independently associated with major bleedings in patients with AF (43), also suggesting higher hemorrhage risk in RB ablation.

Interestingly, results from LASSO logistic regression and random forest presented a different explanation for proteins associated with hemorrhage and coagulation. Among the significant proteins selected in three groups by LASSO logistic regression and random forest, respectively, only one protein in the RF group was associated with hemorrhage and coagulation activity, compared with two related proteins in the CB group and three in the RB group. Although RF ablation affected more proteins associated with thrombus and bleeding events, they are not characteristic enough to distinguish RF from other techniques. The underlying reason for a greater impact on hemorrhage and coagulation activity in balloon-based ablation groups shown in two novel bioinformatic analyses could be attributed to the larger ablation lesion.

In clinical practice, the basis for the selection of ablation technologies for pulmonary vein isolation in patients with AF remains unclear, especially with the emergence of a new technique RB. A recent clinical study showed that all ablation technologies facilitate safe and efficient pulmonary vein isolation, with slight differences in the procedural data and complications (44). To replenish previous clinical studies, the similar alteration of two balloon-based ablations in protein profile in our study provided the pathophysiological evidence from protein levels to support the application of RB in the clinic.

## Limitations

This study was limited to the proteins provided by the Olink panel and the evaluation period. Thus, most of the investigated proteins are still not clearly related to the clinical event, although they are useful for the pathophysiological understanding of the ablation lesion mechanism. Finally, the size of this explorative study was small and, as such, did not have enough power to show minor differences between the three ablation techniques.

## Conclusion

Multiple plasma proteins were differently expressed after catheter ablation using different techniques, especially on the first day after ablation, suggesting that longer monitoring is not needed in patients with AF undergoing uncomplicated catheter ablation. The pattern of the protein change during the acute phase is mainly affected by the lesion size according to the similar enrichment analysis in the most significant module of CB and RB ablation. We also identified some important plasma proteins that could be of potential interest to future studies.

By comparison, balloon-based ablation (CB and RB) showed a higher tendency of inflammation and had more influence on hemorrhage and coagulation activity. Further studies of these biomarkers and clinical events in patients with AF who received different ablations are warranted.

## Data availability statement

The original contributions presented in this study are included in the article/Supplementary material, further inquiries can be directed to the corresponding authors.

## Ethics statement

The studies involving human participants were reviewed and approved by the Ruijin Hospital Ethics Committee. The patients/participants provided their written informed consent to participate in this study.

## Author contributions

ML: data curation-lead, formal analysis-lead, investigation-equal, methodology-equal, validation-equal, visualization-equal, and writing—original draft, review, and editing-equal. YB: conceptualization-lead, investigation-equal, methodology-equal, validation-equal, and writing—original draft, review, and editing-equal. ZD: conceptualization-supporting, data curation-supporting, investigation-equal, methodology-equal, validation-equal, and writing—original draft, review, and editing-equal. NZ, CL, YX, YW, QZL, WP, TL, and QJ: methodology-equal. YYX: conceptualization-supporting and methodology-equal. YZ and RZ: methodology-equal and visualization-equal. QHL: investigation-equal and validation-equal. JQ, TZ, and CX: investigation-equal. YAX, LJW, and LQW: writing—review and editing-equal. TY: conceptualization-supporting, funding acquisition-equal, supervision-equal, and writing—review and editing-equal. YCX: conceptualization-supporting, data curation-supporting, funding acquisition-equal, supervision-equal, and writing—review and editing-equal. All authors contributed to the article and approved the submitted version.
