# Supplementary material for: Plasma protein profiling analysis in patients with atrial fibrillation before and after three different ablation techniques
Source: Front Cardiovasc Med. 2023 Jan 10;9:1077992. doi: 10.3389/fcvm.2022.1077992 (PMC9871787; doi:10.3389/fcvm.2022.1077992)
Supplement: Supplementary file 1 [file Data_Sheet_1.docx]

**Supplemental Materials**

**Supplemental Table S1. The GO_BP terms enriched for the increased proteins involved in the most significant module in the cryoballoon ablation group.**

| Term ID | Term description | False discovery rate |
| --- | --- | --- |
| GO:0019221 | Cytokine-mediated signaling pathway | 1.79E-07 |
| GO:0030595 | Leukocyte chemotaxis | 1.79E-07 |
| GO:0006954 | Inflammatory response | 1.02E-06 |
| GO:0097529 | Myeloid leukocyte migration | 2.50E-06 |
| GO:0002687 | Positive regulation of leukocyte migration | 4.19E-06 |
| GO:0006952 | Defense response | 4.19E-06 |
| GO:0002548 | Monocyte chemotaxis | 4.25E-06 |
| GO:0071310 | Cellular response to organic substance | 6.09E-06 |
| GO:0006955 | Immune response | 1.47E-05 |
| GO:0030335 | Positive regulation of cell migration | 2.32E-05 |
| GO:0030593 | Neutrophil chemotaxis | 2.32E-05 |
| GO:0010647 | Positive regulation of cell communication | 2.65E-05 |
| GO:0023056 | Positive regulation of signaling | 2.65E-05 |
| GO:0070098 | Chemokine-mediated signaling pathway | 2.65E-05 |
| GO:0002690 | Positive regulation of leukocyte chemotaxis | 3.34E-05 |
| GO:0051707 | Response to other organism | 5.55E-05 |
| GO:0006950 | Response to stress | 6.33E-05 |
| GO:0010646 | Regulation of cell communication | 6.64E-05 |
| GO:0023051 | Regulation of signaling | 7.06E-05 |
| GO:0048584 | Positive regulation of response to stimulus | 8.04E-05 |
| GO:0009605 | Response to external stimulus | 9.43E-05 |
| GO:0098542 | Defense response to other organism | 0.00019 |
| GO:0048583 | Regulation of response to stimulus | 0.00021 |
| GO:0071347 | Cellular response to interleukin-1 | 0.00021 |
| GO:0002684 | Positive regulation of immune system process | 0.00024 |
| GO:0009967 | Positive regulation of signal transduction | 0.00024 |
| GO:0032879 | Regulation of localization | 0.00028 |
| GO:0032101 | Regulation of response to external stimulus | 0.00031 |
| GO:0001934 | Positive regulation of protein phosphorylation | 0.00032 |
| GO:0048247 | Lymphocyte chemotaxis | 0.00032 |
| GO:1901701 | Cellular response to oxygen-containing compound | 0.00037 |
| GO:0044093 | Positive regulation of molecular function | 0.0004 |
| GO:0022603 | Regulation of anatomical structure morphogenesis | 0.00043 |
| GO:0071356 | Cellular response to tumor necrosis factor | 0.00056 |
| GO:0009966 | Regulation of signal transduction | 0.00057 |
| GO:0007165 | Signal transduction | 0.00065 |
| GO:0065009 | Regulation of molecular function | 0.00068 |
| GO:0051173 | Positive regulation of nitrogen compound metabolic process | 0.00074 |
| GO:0031325 | Positive regulation of cellular metabolic process | 0.001 |
| GO:0048661 | Positive regulation of smooth muscle cell proliferation | 0.0012 |
| GO:0010604 | Positive regulation of macromolecule metabolic process | 0.0015 |
| GO:0002521 | Leukocyte differentiation | 0.0016 |
| GO:0048522 | Positive regulation of cellular process | 0.0018 |
| GO:0042127 | Regulation of cell population proliferation | 0.0032 |
| GO:0050793 | Regulation of developmental process | 0.0032 |
| GO:0032268 | Regulation of cellular protein metabolic process | 0.0035 |
| GO:0045321 | Leukocyte activation | 0.0035 |
| GO:0051240 | Positive regulation of multicellular organismal process | 0.0046 |
| GO:0048245 | Eosinophil chemotaxis | 0.0049 |
| GO:1902531 | Regulation of intracellular signal transduction | 0.005 |
| GO:0032682 | Negative regulation of chemokine production | 0.0051 |
| GO:1902533 | Positive regulation of intracellular signal transduction | 0.0053 |
| GO:0071346 | Cellular response to interferon-gamma | 0.0054 |
| GO:0071396 | Cellular response to lipid | 0.0068 |
| GO:0043410 | Positive regulation of mapk cascade | 0.0074 |
| GO:0030225 | Macrophage differentiation | 0.0076 |
| GO:0071222 | Cellular response to lipopolysaccharide | 0.0076 |
| GO:1900120 | Regulation of receptor binding | 0.0076 |
| GO:2000026 | Regulation of multicellular organismal development | 0.0099 |
| GO:0070374 | Positive regulation of erk1 and erk2 cascade | 0.0103 |
| GO:0043393 | Regulation of protein binding | 0.0107 |
| GO:0032944 | Regulation of mononuclear cell proliferation | 0.0116 |
| GO:0009617 | Response to bacterium | 0.0122 |
| GO:1904707 | Positive regulation of vascular associated smooth muscle cell proliferation | 0.0122 |
| GO:2000403 | Positive regulation of lymphocyte migration | 0.014 |
| GO:0007566 | Embryo implantation | 0.016 |
| GO:0008285 | Negative regulation of cell population proliferation | 0.0167 |
| GO:0045087 | Innate immune response | 0.0172 |
| GO:0045124 | Regulation of bone resorption | 0.0172 |
| GO:0030155 | Regulation of cell adhesion | 0.0178 |
| GO:0006959 | Humoral immune response | 0.0204 |
| GO:0042742 | Defense response to bacterium | 0.0206 |
| GO:0071622 | Regulation of granulocyte chemotaxis | 0.0228 |
| GO:0043030 | Regulation of macrophage activation | 0.0259 |
| GO:0045765 | Regulation of angiogenesis | 0.0259 |
| GO:1903532 | Positive regulation of secretion by cell | 0.0278 |
| GO:0048545 | Response to steroid hormone | 0.0315 |
| GO:0048585 | Negative regulation of response to stimulus | 0.0325 |
| GO:0002637 | Regulation of immunoglobulin production | 0.0379 |
| GO:0043687 | Post-translational protein modification | 0.039 |
| GO:0014015 | Positive regulation of gliogenesis | 0.0396 |
| GO:0008284 | Positive regulation of cell population proliferation | 0.0403 |
| GO:0080090 | Regulation of primary metabolic process | 0.0419 |

**Supplemental Table S2. The GO_BP terms enriched for the decreased proteins involved in the most significant module in the cryoballoon ablation group.**

| Term ID | Term description | False discovery rate |
| --- | --- | --- |
| GO:0030593 | Neutrophil chemotaxis | 2.05E-13 |
| GO:0031640 | Killing of cells of other organism | 2.05E-13 |
| GO:0070098 | Chemokine-mediated signaling pathway | 2.05E-13 |
| GO:0061844 | Antimicrobial humoral immune response mediated by antimicrobial peptide | 3.89E-13 |
| GO:0048247 | Lymphocyte chemotaxis | 2.87E-12 |
| GO:0006954 | Inflammatory response | 6.24E-09 |
| GO:0006874 | Cellular calcium ion homeostasis | 5.11E-07 |
| GO:0002548 | Monocyte chemotaxis | 5.22E-07 |
| GO:0007186 | G protein-coupled receptor signaling pathway | 1.64E-06 |
| GO:0098542 | Defense response to other organism | 1.64E-05 |
| GO:0051282 | Regulation of sequestering of calcium ion | 2.01E-05 |
| GO:0071346 | Cellular response to interferon-gamma | 3.98E-05 |
| GO:0071347 | Cellular response to interleukin-1 | 5.29E-05 |
| GO:0051281 | Positive regulation of release of sequestered calcium ion into cytosol | 8.22E-05 |
| GO:0002685 | Regulation of leukocyte migration | 9.49E-05 |
| GO:0070374 | Positive regulation of erk1 and erk2 cascade | 9.49E-05 |
| GO:0071356 | Cellular response to tumor necrosis factor | 0.00017 |
| GO:0009615 | Response to virus | 0.00032 |
| GO:0007204 | Positive regulation of cytosolic calcium ion concentration | 0.00033 |
| GO:0043547 | Positive regulation of gtpase activity | 0.001 |
| GO:0007189 | Adenylate cyclase-activating g protein-coupled receptor signaling pathway | 0.0019 |
| GO:0007267 | Cell-cell signaling | 0.002 |
| GO:0002687 | Positive regulation of leukocyte migration | 0.0021 |
| GO:0010818 | T cell chemotaxis | 0.0022 |
| GO:0048245 | Eosinophil chemotaxis | 0.0032 |
| GO:0071222 | Cellular response to lipopolysaccharide | 0.004 |
| GO:1901739 | Regulation of myoblast fusion | 0.004 |
| GO:0001932 | Regulation of protein phosphorylation | 0.0055 |
| GO:0032879 | Regulation of localization | 0.0059 |
| GO:0042127 | Regulation of cell population proliferation | 0.0087 |
| GO:0033993 | Response to lipid | 0.0128 |
| GO:0048522 | Positive regulation of cellular process | 0.0159 |
| GO:0051050 | Positive regulation of transport | 0.016 |
| GO:0045661 | Regulation of myoblast differentiation | 0.0172 |
| GO:0048584 | Positive regulation of response to stimulus | 0.0321 |
| GO:0050790 | Regulation of catalytic activity | 0.0404 |
| GO:0002690 | Positive regulation of leukocyte chemotaxis | 0.046 |

**Supplemental Table S3. The GO_BP terms enriched for the increased proteins involved in the most significant module in the radiofrequency balloon ablation group.**

| Term ID | Term description | | False discovery rate |
| --- | --- | --- | --- |
| GO:0010647 | | Positive regulation of cell communication | 0.0085 |
| GO:0023056 | | Positive regulation of signaling | 0.0085 |
| GO:0001934 | | Positive regulation of protein phosphorylation | 0.0093 |
| GO:0071310 | | Cellular response to organic substance | 0.0093 |
| GO:0071345 | | Cellular response to cytokine stimulus | 0.0093 |
| GO:0043410 | | Positive regulation of mapk cascade | 0.0101 |
| GO:0032740 | | Positive regulation of interleukin-17 production | 0.0129 |
| GO:0006955 | | Immune response | 0.016 |
| GO:0009967 | | Positive regulation of signal transduction | 0.016 |
| GO:0019221 | | Cytokine-mediated signaling pathway | 0.016 |
| GO:0031325 | | Positive regulation of cellular metabolic process | 0.016 |
| GO:0051173 | | Positive regulation of nitrogen compound metabolic process | 0.016 |
| GO:0002675 | | Positive regulation of acute inflammatory response | 0.0173 |
| GO:0010604 | | Positive regulation of macromolecule metabolic process | 0.0173 |
| GO:0051240 | | Positive regulation of multicellular organismal process | 0.0173 |
| GO:1902531 | | Regulation of intracellular signal transduction | 0.0173 |
| GO:0070741 | | Response to interleukin-6 | 0.0212 |
| GO:0007566 | | Embryo implantation | 0.0244 |
| GO:0045321 | | Leukocyte activation | 0.0244 |
| GO:0045124 | | Regulation of bone resorption | 0.0254 |
| GO:2000026 | | Regulation of multicellular organismal development | 0.0254 |
| GO:0048583 | | Regulation of response to stimulus | 0.0272 |
| GO:0002521 | | Leukocyte differentiation | 0.0301 |
| GO:0042531 | | Positive regulation of tyrosine phosphorylation of stat protein | 0.0482 |

**Supplemental Table S4. The GO_BP terms enriched for the decreased proteins involved in the most significant module in the radiofrequency balloon ablation group.**

| Term ID | Term description | False discovery rate | |
| --- | --- | --- | --- |
| GO:0030593 | Neutrophil chemotaxis | | 1.63E-12 |
| GO:0070098 | Chemokine-mediated signaling pathway | 1.63E-12 | |
| GO:0006959 | Humoral immune response | 2.78E-12 | |
| GO:0061844 | Antimicrobial humoral immune response mediated by antimicrobial peptide | 3.10E-12 | |
| GO:0006954 | Inflammatory response | 2.26E-10 | |
| GO:0031640 | Killing of cells of other organism | 3.01E-10 | |
| GO:0019221 | Cytokine-mediated signaling pathway | 1.76E-09 | |
| GO:0048247 | Lymphocyte chemotaxis | 5.47E-09 | |
| GO:0051707 | Response to other organism | 1.52E-07 | |
| GO:0007186 | G protein-coupled receptor signaling pathway | 1.27E-05 | |
| GO:0002687 | Positive regulation of leukocyte migration | 6.72E-05 | |
| GO:0098542 | Defense response to other organism | 7.59E-05 | |
| GO:0006874 | Cellular calcium ion homeostasis | 9.28E-05 | |
| GO:0071346 | Cellular response to interferon-gamma | 9.28E-05 | |
| GO:0071347 | Cellular response to interleukin-1 | 0.00012 | |
| GO:0071222 | Cellular response to lipopolysaccharide | 0.00014 | |
| GO:0002548 | Monocyte chemotaxis | 0.00016 | |
| GO:0030335 | Positive regulation of cell migration | 0.00017 | |
| GO:0071356 | Cellular response to tumor necrosis factor | 0.00034 | |
| GO:0009615 | Response to virus | 0.00063 | |
| GO:0002682 | Regulation of immune system process | 0.00091 | |
| GO:0002690 | Positive regulation of leukocyte chemotaxis | 0.0012 | |
| GO:0033993 | Response to lipid | 0.0013 | |
| GO:0042127 | Regulation of cell population proliferation | 0.0014 | |
| GO:0042592 | Homeostatic process | 0.0015 | |
| GO:0002684 | Positive regulation of immune system process | 0.002 | |
| GO:0032101 | Regulation of response to external stimulus | 0.0026 | |
| GO:0051282 | Regulation of sequestering of calcium ion | 0.0026 | |
| GO:0010818 | T cell chemotaxis | 0.0027 | |
| GO:0051709 | Regulation of killing of cells of other organism | 0.0027 | |
| GO:1904064 | Positive regulation of cation transmembrane transport | 0.0031 | |
| GO:0001775 | Cell activation | 0.0032 | |
| GO:0032103 | Positive regulation of response to external stimulus | 0.0039 | |
| GO:0048245 | Eosinophil chemotaxis | 0.0039 | |
| GO:0007267 | Cell-cell signaling | 0.0041 | |
| GO:0048584 | Positive regulation of response to stimulus | 0.0064 | |
| GO:0090023 | Positive regulation of neutrophil chemotaxis | 0.0065 | |
| GO:0043372 | Positive regulation of cd4-positive, alpha-beta t cell differentiation | 0.0078 | |
| GO:0070374 | Positive regulation of erk1 and erk2 cascade | 0.0078 | |
| GO:0043903 | Regulation of symbiotic process | 0.0096 | |
| GO:0001932 | Regulation of protein phosphorylation | 0.0112 | |
| GO:0065008 | Regulation of biological quality | 0.0112 | |
| GO:0048583 | Regulation of response to stimulus | 0.0124 | |
| GO:0032735 | Positive regulation of interleukin-12 production | 0.0128 | |
| GO:0051281 | Positive regulation of release of sequestered calcium ion into cytosol | 0.0139 | |
| GO:0051345 | Positive regulation of hydrolase activity | 0.015 | |
| GO:1901700 | Response to oxygen-containing compound | 0.015 | |
| GO:0032879 | Regulation of localization | 0.016 | |
| GO:0007204 | Positive regulation of cytosolic calcium ion concentration | 0.0177 | |
| GO:0032731 | Positive regulation of interleukin-1 beta production | 0.0213 | |
| GO:0045321 | Leukocyte activation | 0.0264 | |
| GO:0051050 | Positive regulation of transport | 0.0264 | |
| GO:0019932 | Second-messenger-mediated signaling | 0.0266 | |
| GO:0010959 | Regulation of metal ion transport | 0.0351 | |
| GO:0001934 | Positive regulation of protein phosphorylation | 0.0361 | |
| GO:0060341 | Regulation of cellular localization | 0.037 | |
| GO:0043547 | Positive regulation of gtpase activity | 0.0382 | |
| GO:0002697 | Regulation of immune effector process | 0.0407 | |
| GO:0032642 | Regulation of chemokine production | 0.0417 | |
| GO:0032760 | Positive regulation of tumor necrosis factor production | 0.0449 | |

**Supplemental Table S5. The GO_BP terms enriched for the increased proteins involved in the most significant module in the radiofrequency ablation group.**

| Term ID | Term description | False discovery rate |
| --- | --- | --- |
| GO:0022603 | Regulation of anatomical structure morphogenesis | 0.0048 |
| GO:0032101 | Regulation of response to external stimulus | 0.0048 |
| GO:0002690 | Positive regulation of leukocyte chemotaxis | 0.0055 |
| GO:0006952 | Defense response | 0.0055 |
| GO:0006954 | Inflammatory response | 0.0062 |
| GO:0030595 | Leukocyte chemotaxis | 0.0072 |
| GO:0010647 | Positive regulation of cell communication | 0.0091 |
| GO:0019221 | Cytokine-mediated signaling pathway | 0.0091 |
| GO:0023056 | Positive regulation of signaling | 0.0091 |
| GO:0032682 | Negative regulation of chemokine production | 0.0113 |
| GO:0032944 | Regulation of mononuclear cell proliferation | 0.0115 |
| GO:0098542 | Defense response to other organism | 0.0147 |
| GO:0009605 | Response to external stimulus | 0.0148 |
| GO:0002684 | Positive regulation of immune system process | 0.016 |
| GO:0071310 | Cellular response to organic substance | 0.016 |
| GO:1901701 | Cellular response to oxygen-containing compound | 0.0216 |
| GO:1903532 | Positive regulation of secretion by cell | 0.0216 |
| GO:0048545 | Response to steroid hormone | 0.0217 |
| GO:0002521 | Leukocyte differentiation | 0.0219 |
| GO:0002548 | Monocyte chemotaxis | 0.0219 |
| GO:0032879 | Regulation of localization | 0.0219 |
| GO:0045124 | Regulation of bone resorption | 0.0219 |
| GO:0043687 | Post-translational protein modification | 0.0226 |
| GO:0043030 | Regulation of macrophage activation | 0.0267 |
| GO:0045785 | Positive regulation of cell adhesion | 0.0289 |
| GO:0051173 | Positive regulation of nitrogen compound metabolic process | 0.0356 |
| GO:0002637 | Regulation of immunoglobulin production | 0.037 |
| GO:0014015 | Positive regulation of gliogenesis | 0.0383 |
| GO:0031325 | Positive regulation of cellular metabolic process | 0.0427 |
| GO:0002718 | Regulation of cytokine production involved in immune response | 0.0447 |
| GO:0006955 | Immune response | 0.0447 |
| GO:0009967 | Positive regulation of signal transduction | 0.0447 |
| GO:0010604 | Positive regulation of macromolecule metabolic process | 0.0447 |
| GO:0046427 | Positive regulation of receptor signaling pathway via jak-stat | 0.0447 |
| GO:0048661 | Positive regulation of smooth muscle cell proliferation | 0.0447 |
| GO:0070661 | Leukocyte proliferation | 0.0447 |
| GO:0071396 | Cellular response to lipid | 0.0447 |

**
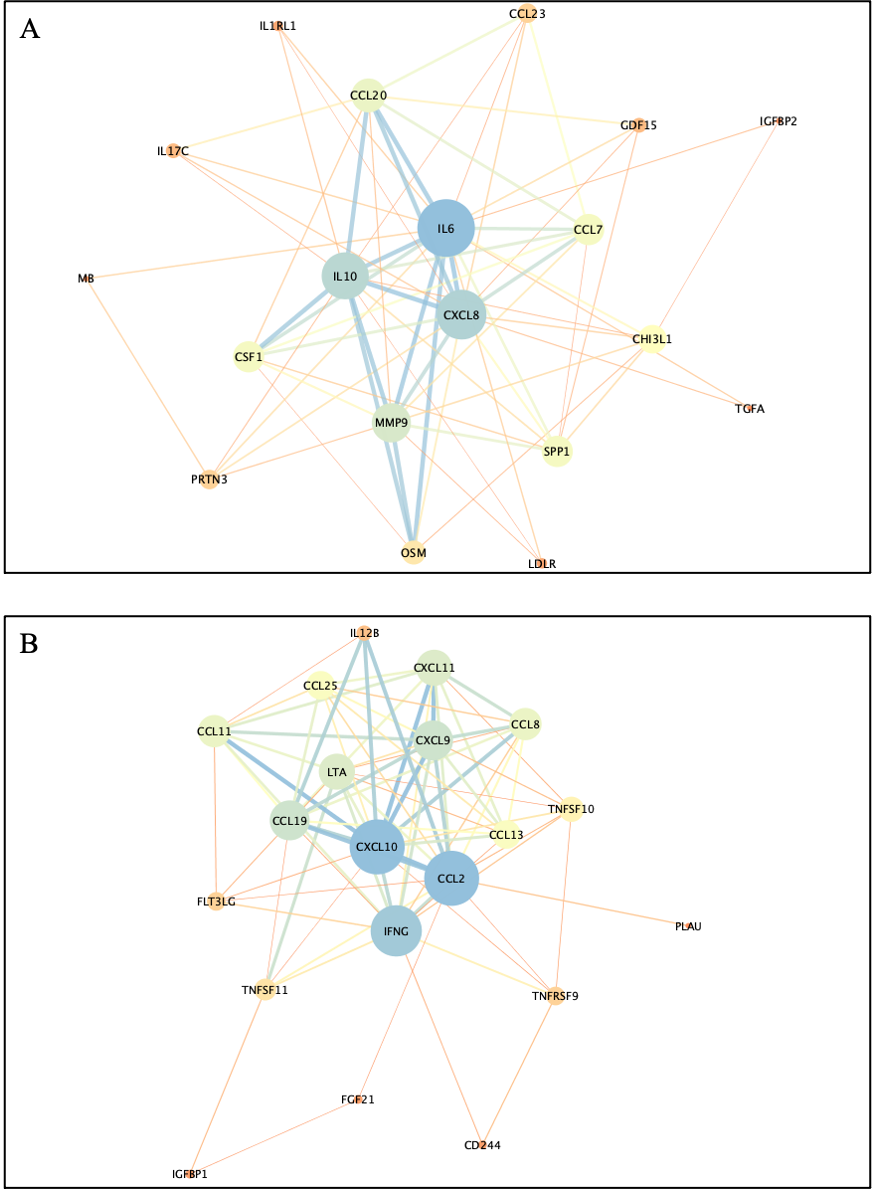
**

**Supplemental Figure S1. PPI network of altered proteins in the cryoballoon group.**

PPI network of the increased proteins (A) and the decreased proteins (B) in CB group. The sizes and colors of the nodes are illustrated from big to small and dark to bright in descending order of degree values. The sizes and colors of the edges are illustrated from big to small and dark to bright in descending order of combined score.


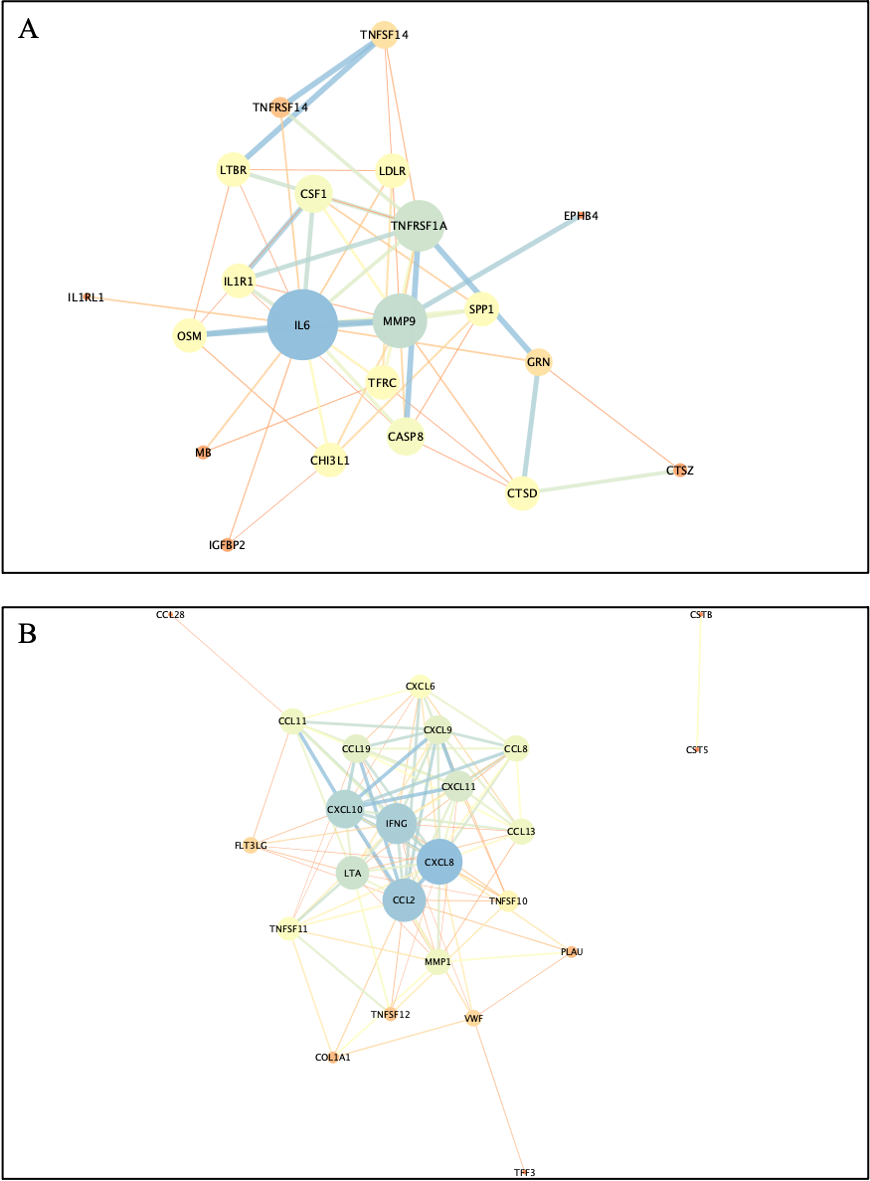


**Supplemental Figure S2. PPI network of altered proteins in the radiofrequency balloon group.**

PPI network of the increased proteins (A) and the decreased proteins (B) in RB group. The sizes and colors of the nodes are illustrated from big to small and dark to bright in descending order of degree values. The sizes and colors of the edges are illustrated from big to small and dark to bright in descending order of combined score.

**
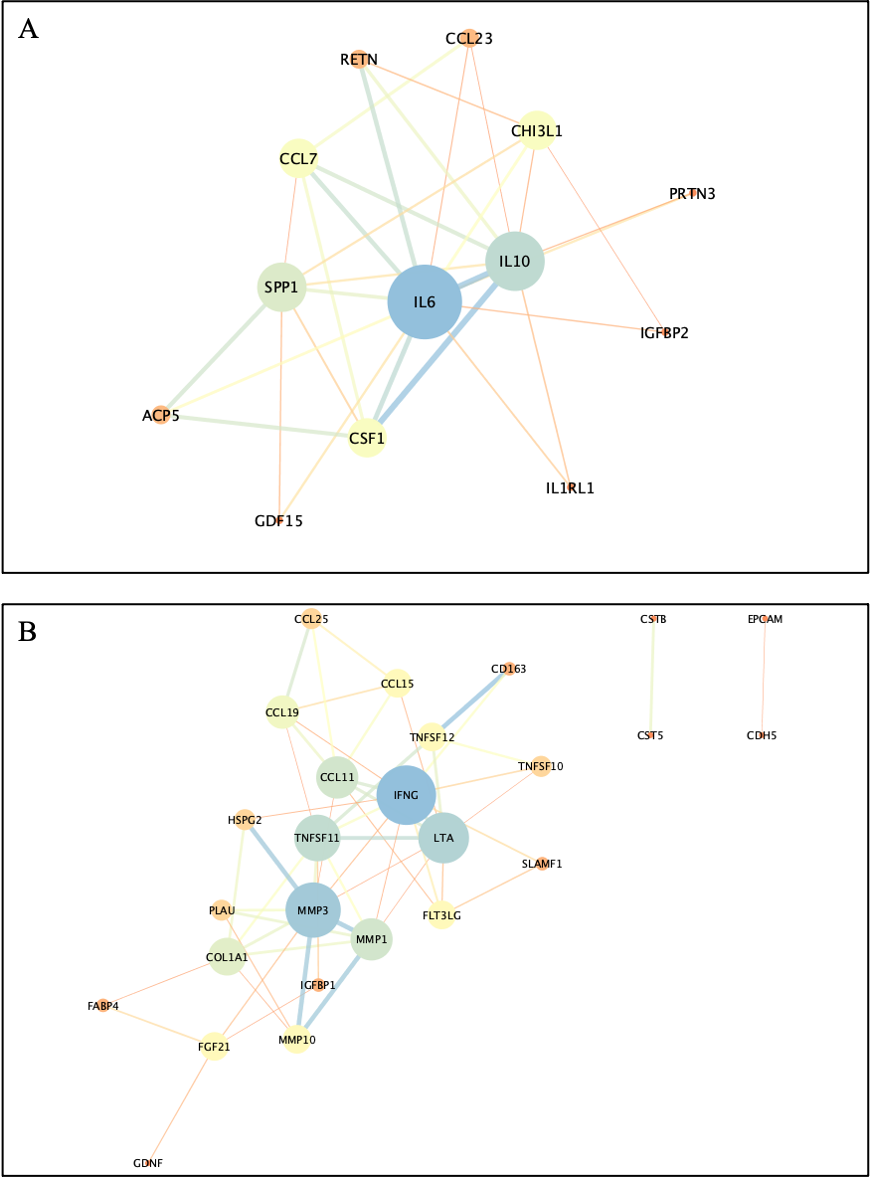
**

**Supplemental Figure S3. PPI network of altered proteins in the radiofrequency group.**

PPI network of the increased proteins (A) and the decreased proteins (B) in RF group. The sizes and colors of the nodes are illustrated from big to small and dark to bright in descending order of degree values. The sizes and colors of the edges are illustrated from big to small and dark to bright in descending order of combined score.


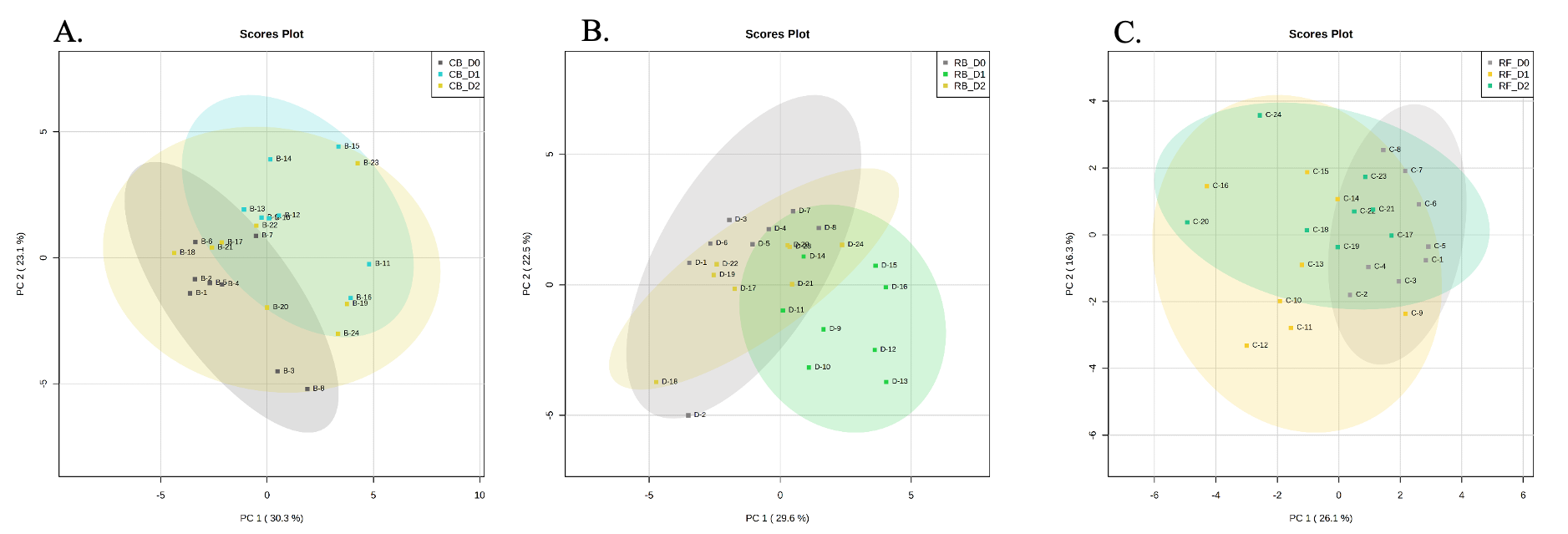


**Supplemental Figure S4. Principle Component Analysis (PCA) diagram of altered proteins before and after ablation.**

PCA diagram of altered proteins before and after ablation in CB group (A), in RB group (B) and in RF group (C).
